# Investigating the synergistic effects of biochar, trans-zeatin riboside, and *Azospirillum brasilense* on soil improvement and enzymatic activity in water-stressed wheat

**DOI:** 10.1186/s12870-024-05038-z

**Published:** 2024-04-23

**Authors:** Muhammad Saqlain Zaheer, Muhammad Rizwan, Nazish Aijaz, Akhtar Hameed, Kamran Ikram, Hafiz Haider Ali, Yasir Niaz, Hafiz M. Usman Aslam, Salim Manoharadas, Muhammad Waheed Riaz, Nadeem Ahmed, Rani Bibi, Muhammad Aamir Manzoor, Shamsur Rehman

**Affiliations:** 1 Department of Agricultural Engineering, Khwaja Fareed University of Engineering and Information Technology, Rahim Yar Khan, Pakistan; 2 Department of Plant Nutrition, Institute of Crop Science and Resource Conservation (INRES), University of Bonn, 53115 Bonn, Germany; 3 School of Biomedical Science, Hunan University, Changsha, Hunan China; 4 MOA Key Laboratory of Soil Microbiology, Rhizobium Research Center, China Agricultural University, Beijing, China; 5 Institute of Plant Protection, MNS University of Agriculture, Multan, 61000 Pakistan; 6 Department of Agriculture, Government College University, Lahore, 54000 Pakistan; 7 Crop, Soil, and Environmental Sciences, University of Arkansas, Fayetteville, AR USA; 8 Department of Botany and Microbiology, College of Science, King Saud University, Riyadh, 11451 Saudi Arabia; 9 State Key Laboratory of Wheat Breeding, Group of Wheat Quality and Molecular Breeding, College of Agronomy, Shandong Agricultural University, Tai’an, Shandong 271000 China; 10 Department of Plant Science, School of Agriculture and Biology, Shanghai Jiao Tong University, Shanghai, P.R. China; 11 National Key Laboratory of Wheat Improvement, Peking University Institute of Advanced Agricultural Sciences, Weifang, 261325 China; 12 Department of Plant Pathology, San Luis Valley Research Center, Colorado State University, Colorado, USA

**Keywords:** Biochar, Trans-zeatin riboside, *Azospirillum brasilense*, Soil improvement, Soil amendments enzymatic activity, Drought, Plant physiology

## Abstract

**Background:**

Water stress is a major danger to crop yield, hence new approaches to strengthen plant resilience must be developed. To lessen the negative effects of water stress on wheat plants, present study was arranged to investigate the role of synergistic effects of biochar, trans-zeatin riboside (t-ZR), and *Azospirillum brasilense* on soil improvement and enzymatic activity in water-stressed wheat.

**Results:**

In a three-replication experiment comprising of four treatments (T_0_: Control, T_1_: Drought stress (DS), T_2_: DS + t-ZR with biochar, T_3_: DS + *A. brasilense* with biochar), we observed notable improvements in soil quality and enzymatic activities in water-stressed wheat plants with the application of t-ZR and *A. brasilense* with biochar. In drought stress, Treatment having the application of *A. brasilense* with biochar performs best as compared to the other and significant increased the enzymatic activities such as peroxidase (7.36%), catalase (8.53%), superoxide dismutase (6.01%), polyphenol oxidase (14.14%), and amylase (16.36%) in wheat plants. Different enzymatic activities showed different trends of results. Soil organic C, dissolved organic C, dissolved organic N also enhanced 29.46%, 8.59%, 22.70% respectively with the application of *A. brasilense* with biochar under drought stress condition.

**Conclusions:**

The synergistic action of *A. brasilense* and biochar creates an effective microbiological environment that supports essential plant physiological processes during drought stress. This enhancement is attributed to improved soil fertility and increased organic matter content, highlighting the potential of these novel strategies in mitigating water stress effects and enhancing crop resilience.

**Supplementary Information:**

The online version contains supplementary material available at 10.1186/s12870-024-05038-z.

## Introduction

Wheat (*Triticum aestivum* L.) has much greater significance beyond its use as a staple grain. It has greatly influenced the development of culture and economy, fostering the emergence of modern societies and the flourishing of civilizations [1]. Wheat is a symbol of durability and a testament to agricultural human ingenuity because of its adaptability and persistence. Wheat is important because it is a major component of the world’s food supply and grows well in a variety of environments [2]. It is the need to enhance the wheat production on sustainable basis under the current climatic change and the growing global population scenario [3]. Lack of water, extreme heat, and irregular rain patterns can all negatively impact the amount of wheat production [4, 5]. These challenges require a thorough understanding of the mechanisms underlying drought stress in wheat in addition to the development of innovative mitigation strategies [6].

Drought stress poses a serious threat to crop yield worldwide [7]. It is a major concern in the agricultural sector for causing food insecurity. Wheat crop is very crucial for global nutrition and has been significantly harmed by environmental challenges [4]. The twenty-first century is bringing with it more and more clear evidence of climate change, which is increasing the frequency and intensity of droughts [8]. Wheat plant and soil enzymatic activity plays a critical role in determining the overall health and productivity of these essential agricultural components. It’s still unclear how these enzyme activities relate to the way they respond to drought stress [9]. To better understand how plants withstand dry conditions and develop strategies to mitigate the detrimental effects of water scarcity on agricultural output, we must gain insight into how drought stress affects these enzyme functions [10]. Plants under drought stress exhibit a number of physiological and biochemical reactions as they adjust to the shortage of water. Stomatal closure is one of the main physiological effect [7]. It minimizes water loss through transpiration but also reduces the amount of carbon dioxide that can be absorbed for photosynthesis, which lowers growth rates [4]. Drought stress can also result in dehydration of the cells, which can lower turgor pressure, hinder cell development, and eventually lower plant production. Superoxide dismutase (SOD), catalase (CAT), and peroxidase (POX) are among the antioxidant enzymes that effected when plants under drought stress and can cause the production of reactive oxygen species (ROS) [4, 7, 9]. These enzymes stop oxidative stress-induced cellular damage by scavenging ROS. The activity of osmolyte synthesis-related enzymes such as proline and glycine betaine, which support cellular osmotic balance and stabilize proteins and membranes in water-deficient environments, is similarly impacted by drought stress. All the physiological and enzymatic activities severally affected by the drought stress [7, 9, 10].

Biochar has become known as an attractive and promising tool for improving soil health and increasing crop yields, especially in the growing field of environmental science and sustainable agriculture [6]. Biochar is a kind of charcoal generated from the pyrolysis of organic materials that are used to increase plant growth and soil fertility. This is also very important for sustainable agriculture and soil management [11, 12]. Biochar affects soil enzymes, growth, and development of plants; especially in wheat is one of the most fascinating aspects of this material [6]. The enzymatic activity of the soil is essential for the breakdown of organic matter, nutrient cycling, and overall soil health. Adding biochar to soil has been found to impact this enzymatic activity in multiple ways [13, 14]. Biochar can also change the makeup of the microbial population which affects soil enzymes both directly and indirectly in addition to increasing nutrient availability and soil organic carbon stability [6]. Understanding the intricate relationship between soil enzymatic activity and biochar is essential to identifying the underlying mechanisms and improving soil conditions to support sustainable agriculture. The effect of biochar on different plant enzymatic processes, including those connected to nutrition intake, photosynthesis, and stress responses, is a topic of growing interest for scientists and experts in agriculture. They are excited to investigate the potential benefits of biochar on wheat growth and resilience, given the crucial role wheat plays in the world food chain [6, 11].

Trans-zeatin riboside (t-ZR) is a naturally occurring plant growth regulator that has emerged as a strong growth hormone used for improving plant growth under stress conditions [15]. This material is a derivative of cytokinin and is necessary for the growth and development of plants [16]. t-ZR has demonstrated promise in reducing the negative effects of stress on plants providing a useful instrument for enhancing plant health and productivity in difficult environmental circumstances. t-ZR functions as a signaling molecule that coordinates different aspects of plant growth and development by influencing these internal processes [15]. t-ZR can contribute to improved nutrient uptake, photosynthesis, and metabolic processes in the plant. It plays a role in encouraging cell elongation and division, both of which support general growth. This enhances plant resilience and productivity, especially when faced with stressors [15, 16].

*Azospirillum brasilense* offers unique chances to enhance soil health and agricultural productivity. Due to its role in increasing enzymatic activity such as nitrogenase, protease, phosphatase, cellulose, lipase activity within the soil ecosystem and nutrient availability for plants, especially wheat, this beneficial soil bacterium has gained recognition [15]. Enzymes are essential to the breakdown of organic materials in soil and the cycling of nutrients and These enzymatic activities are a key sign of the soil’s fertility and health because they greatly influence plant growth and development [17]. *A. brasilense* has demonstrated potential in increasing soil enzymatic activity by controlling the microbial community, nutrient availability, and organic matter breakdown due to its capacity to establish mutualistic relationships with plants. These effects promote improved soil quality and plant growth [15].

Our objective for this study is to highlight the potential of biochar in combination with t-ZR and *A. brasilense* for sustainable wheat farming in water-scarce and ecologically challenging environments. The purpose of this study was to examine how biochar acts in conjunction with t-ZR and *A. brasilense* and how much they can reduce the negative effects of drought stress on soil enzymatic activities in wheat plants. We hypothesized that the application of t-ZR and *A. brasilense* can enhcan enzamatic activities in the soil as well as in the plant. This study is essential for securing a stable and resilient future of wheat production for sustainable agriculture and food security. The use of t-ZR or *A. brasilense* with biochar also provided the future direction that which one is more efficient for soil fertility and to improve plant growth under drought.

## Materials and methods

### Plant growth conditions and experimental layout

A well-planned experiment was conducted at the agricultural research farm, Khwaja Fareed University E&IT, Rahim Yar Khan, Pakistan to investigate the effect of biochar with trans-zeatin riboside (tZR) and *Azospirillum brasilense* under drought stress conditions. Drought stress was applied at the booting stage (after 75 days of sowing) by noting applying the irrigation at the booting stage and data was collected at the anthesis stage (after 100 days of sowing). The experiment consisted of four treatments (T_0_: Control (C) (No drought stress, No application of biochar, t-ZR or *A. brasilense*), T_1_: Drought stress (DS), T_2_: DS + t-ZR with biochar, T_3_: DS + *A. brasilense* with biochar) having three replications. The plot size during this experiment was 5 × 7 ft. “Dilkash 2020” an approved variety of wheat was obtained from the Regional Agricultural Research Institute and the wheat seed was sowed on 20th November, 2022. t-ZR was obtained from the lab and applied @ 25 mg/L, as foliar spray in solution form after 75 days of sowing when drought stress was applied at the booting stage. t-ZR applied only one time during the drought stress condition on wheat crop. *A. brasilense* strains was collected from the GC University Lahore and was inoculated with the wheat seeds at sowing time according to the procedure reported by Fukami et al. [18]. Biochar 9000 kg/ha (pyrolysis at 700◦C) was applied before 5 days of the sowing in the soil with soil preparation according to the treatments (Table 1). All other inputs remained the same for all treatments. Average monthly temperature and humidity of the experimental area during the experiment is shown in Table 2. Four irrigations were applied as per recommendations of agriculture department, 1st on thiilering stage, 2nd on booting, 3rd on flowering and 4th on anthesis stage. Fertilizers (NPK; 120–8060 kg/ha) was also applied as per recomdadations with irrigation water. All other inputs kept same.

**Table 1:** Properties of biochar applied

Biochar Property	Values
pH	10.3
Electric conductivity (µS cm^− 1^)	928
TC (%)	65.2
TN (%)	0.55
Dichromate oxidizable organic C	4.62
Water Soluble Carbon	142
Water Soluble Nitrogen	92.21
Ash content (%)	3.41
Carbonates content (% CaCO_3_)	12.55
CEC (cmol_c_ kg^− 1^)	33.31
NH_4_-N sorption (mg NH_4_- N g^− 1^)	2.23
NO_3_-N sorption (mg NO_3_- N g^− 1^)	N.S.

**Table 2:** Average monthly temperature and humidity during experiment at experimental area

Month	Temperature(°C)	Humidity (%)
November	21	62
December	15	72
January	13	73
February	14	66
March	21	63
April	28	47

### Recorded parameters

To record the soil and plant eyzamatic parameters, All soil and plant samples were obtained at maturity of wheat crop. Homogenate of plant tissue was prepared to determine the peroxidase activities in wheat plants combining it with a peroxidase substrate (guaiacol). By tracking the change in absorbance at a particular wavelength over time, the reaction would be observed. The peroxidase activities were noticed using a standard curve [19]. The process for measuring catalase activity entails incubating the plant extract with hydrogen peroxide and tracking how quickly the hydrogen peroxide breaks down as reported by Hadwan [20]. This was tracked by looking at changes in absorbance at a particular wavelength or the evolution of oxygen. A standard curve was created with known catalase concentrations used for calculations. Superoxide dismutase (SOD) activities were measured by assessing the enzyme’s ability to inhibit a reaction involving superoxide radicals. The spectrophotometric method was employed for this purpose, and commercially available assay kits were used to quantify SOD activity in wheat plants [21]. The oxidation of a phenolic substrate was used to measure polyphenol oxidase (PPO) activity. A standard curve with known enzyme concentrations was used to calculate PPO activity as reported by Yokotsuka et al. [22].

The process for measuring amylase activity entails soaking the plant extract in a starch substrate and watching for the release of reducing sugars [23] and the plant extract is incubated with a protein substrate to determine protease activity [24]. Plant extracts are incubated with a lipid substrate, cellulose substrate, phenylalanine substrate and phosphate substrate, phosphate substrate under alkaline conditions to measure lipase activity, fatty acids, cellulase activity, phenylalanine ammonia-lyase (PAL), acid phosphatase activity and alkaline phosphatase activity respectively. Calculations were made by using a standard curve [25–29]. Nitrate solution was incubated in the plant tissue to evaluate the activity of nitrate reductase, and the amount of nitrate that was converted to nitrite was then measured. The final measurement of nitrate reductase activity was expressed in micromoles per hour per gram of fresh weight, which is obtained by comparing the experimental results to a standard curve with known concentrations [30]. Soil enzymatic activities [urease (UR), dehydrogenase (DH), β-glucosidase (G), and acid phosphatase (AcP)] in the soil were noticed as per procedurereported by Tabatabai [31]. Soil organic carbon (SOC) was measured as procedure reported by Sparks et al. [32] and Dissolved organic nitrogen (DON) and carbon (DOC) were noticed as per procedure described by Smolander and Kitunen [33].

### Statistical analysis

Data was collected from the three replications and arranged in MS Excel – Microsoft for the statistical analysis. Statistical analysis was done with the use of a computer software “Statistix 8.1”. Different alphabetical letters with the data present in tables and figures, shows statistical difference between each treatment.

## Results

**Fig. 1:**
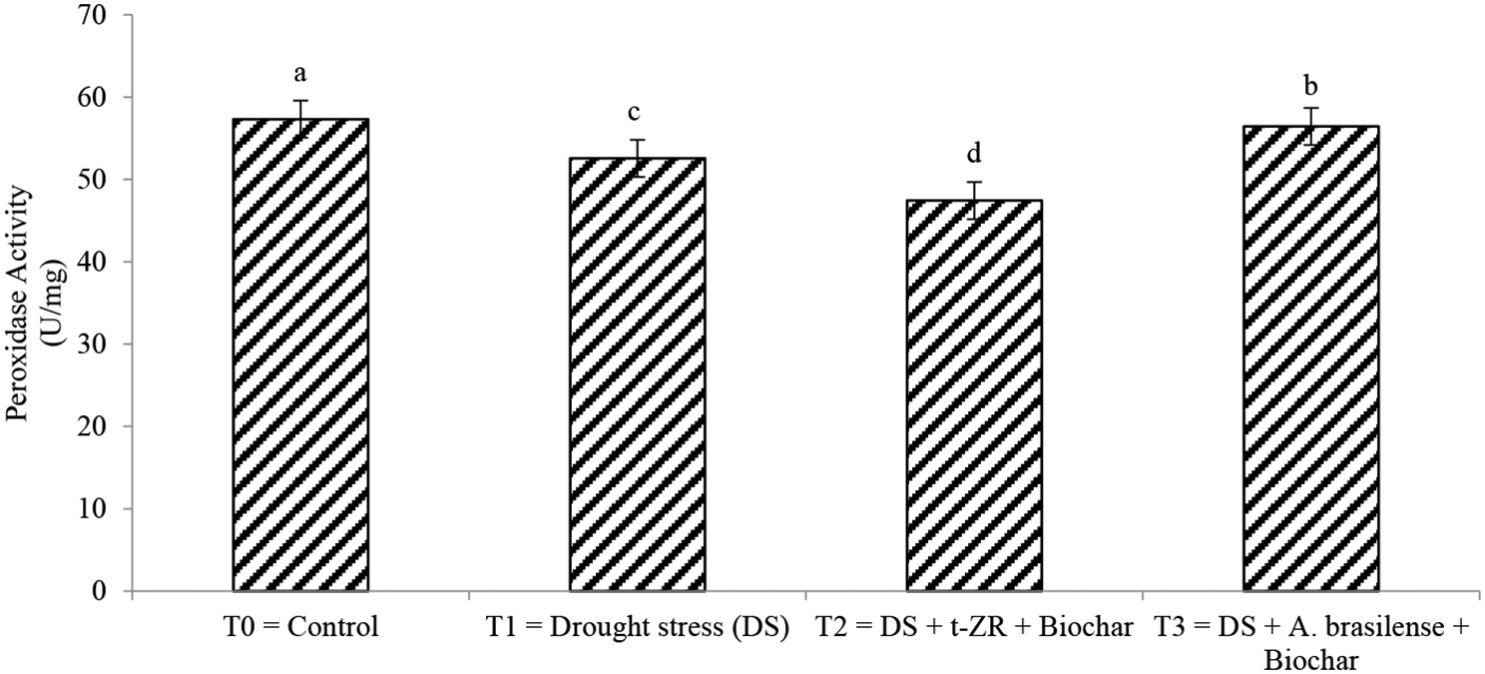
Effect of combine application of biochar with trans-zeatin riboside and with *Azospirillum brasilense* on peroxidase activity (U/mg) of wheat under drought stress

**Fig. 2:**
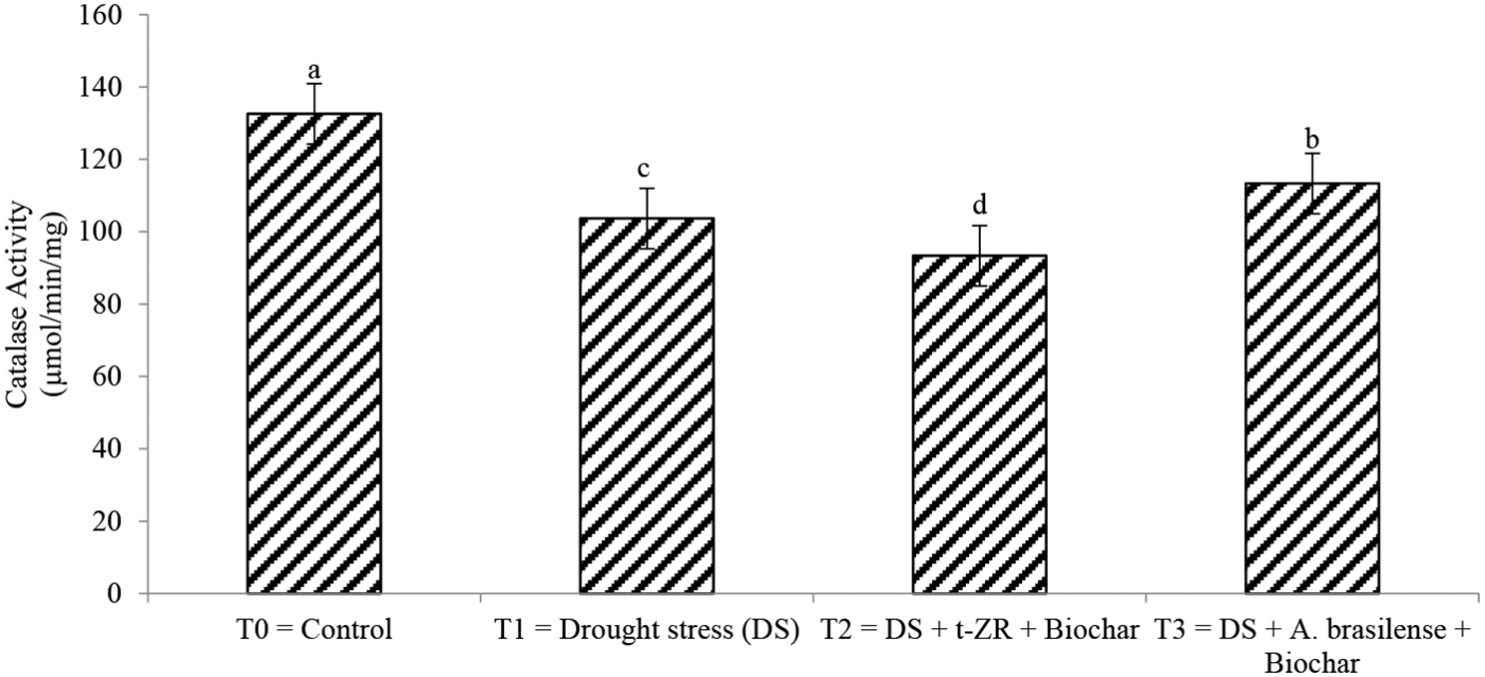
Effect of combine application of biochar with trans-zeatin riboside and with *Azospirillum brasilense* on catalase activity (µmol/min/mg) of wheat under drought stress

Different enzymatic activities affect differently with the application of biochar with trans-zeatin riboside (t-ZR) and biochar with *Azospirillum brasilense* under drought stress. Peroxidase activity and Catalase activity significantly affect all studied treatments (Figs. 1 and 2). The highest peroxidase activity (57.32 U/mg) was noticed in T_0_ having a control condition of no drought stress followed by T_3_ (57.32 U/mg) when biochar was applied with *A. brasilense* under drought stress and lowest peroxidase activities (56.43 U/mg) were noticed in T_2_ when biochar was applied with t-ZR. The highest catalase activities were noticed in T_0_ (132.56 µmol/min/mg) followed by T_4_ (113.32 µmol/min/mg) and the lowest catalase activities (93.34 µmol/min/mg) were seen in T_2_.

**Fig. 3:**
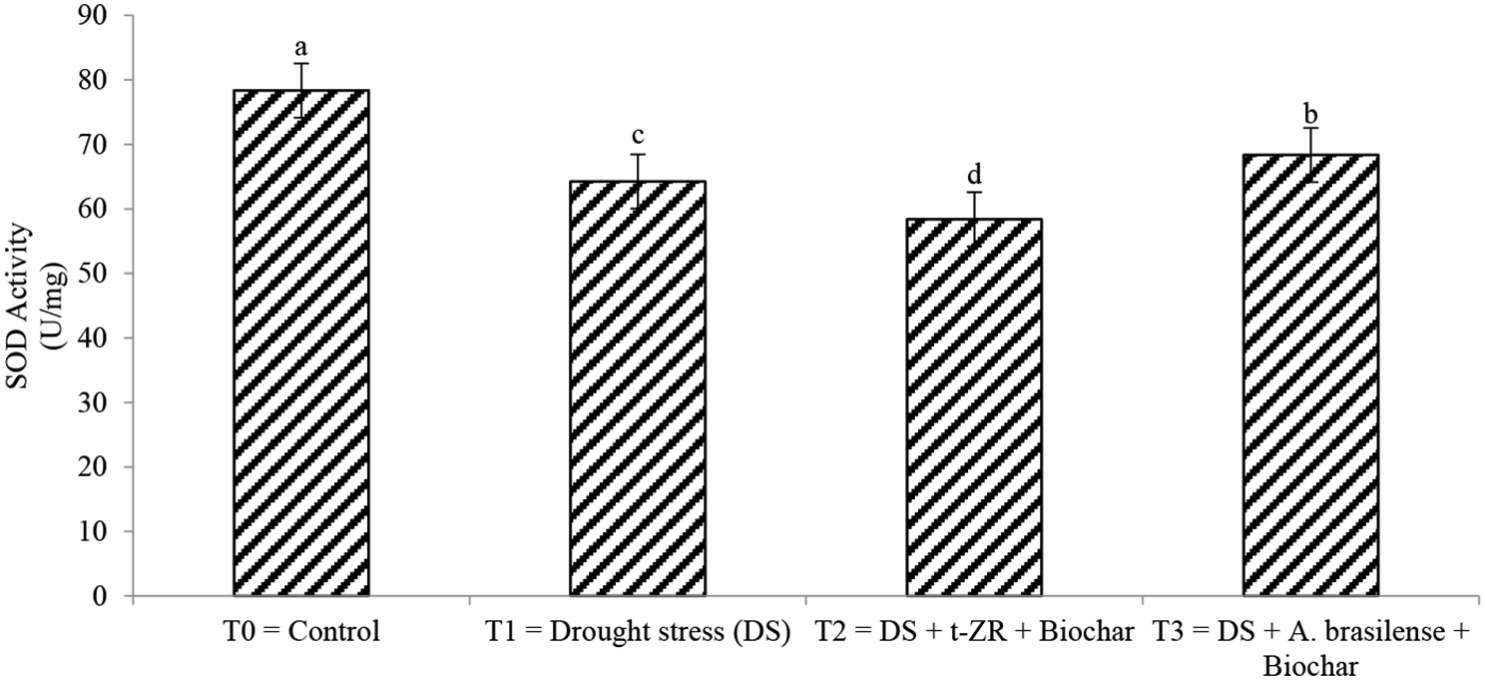
Effect of combine application of biochar with trans-zeatin riboside and with *Azospirillum brasilense* on superoxide dismutase (SOD) (U/mg) of wheat under drought stress

**Fig. 4:**
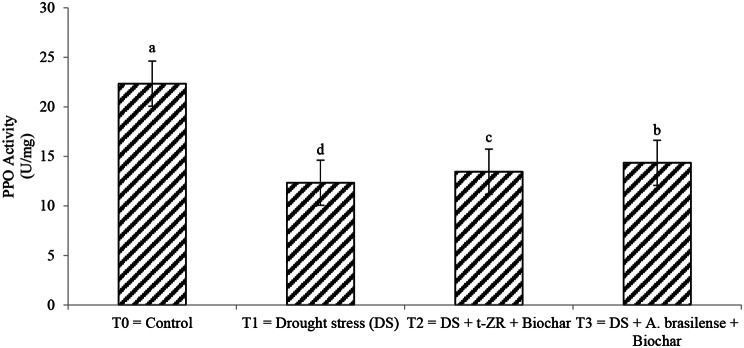
Effect of combine application of biochar with trans-zeatin riboside and with *Azospirillum brasilense* on polyphenoloxidase (PPO) activity (U/mg) of wheat under drought stress

Superoxide dismutase (SOD) and polyphenol oxidase (PPO) were significantly affected by the application of biochar with t-ZR and biochar with *A. brasilense*. The highest SOD activities were noticed in T_0_ (78.34 U/mg) under control treatment having no drought followed by T_4_ (68.34 U/mg) when biochar was applied with *A. brasilense* and the lowest SOD activities were noticed in T_3_ (58.34 U/mg) when biochar was applied with t-ZR (Fig. 3). The highest PPO activities were noticed in T_0_ (22.34 U/mg) followed by T_4_ (14.35 U/mg) and the lowest PPO activities were seen in T_2_ (12.34 U/mg) (Fig. 4).

**Fig. 5:**
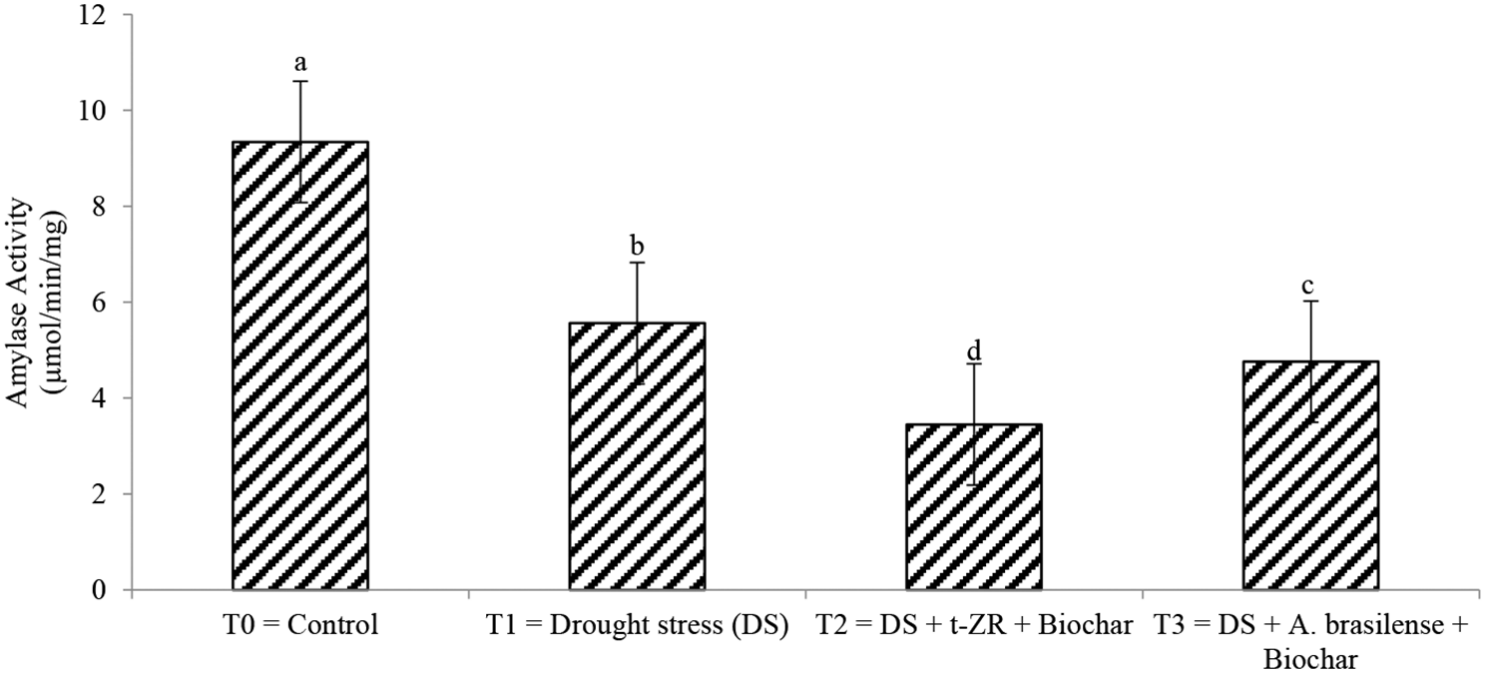
Effect of combine application of biochar with trans-zeatin riboside and with *Azospirillum brasilense* on amylase activity (µmol/min/mg) of wheat under drought stress

**Fig. 6:**
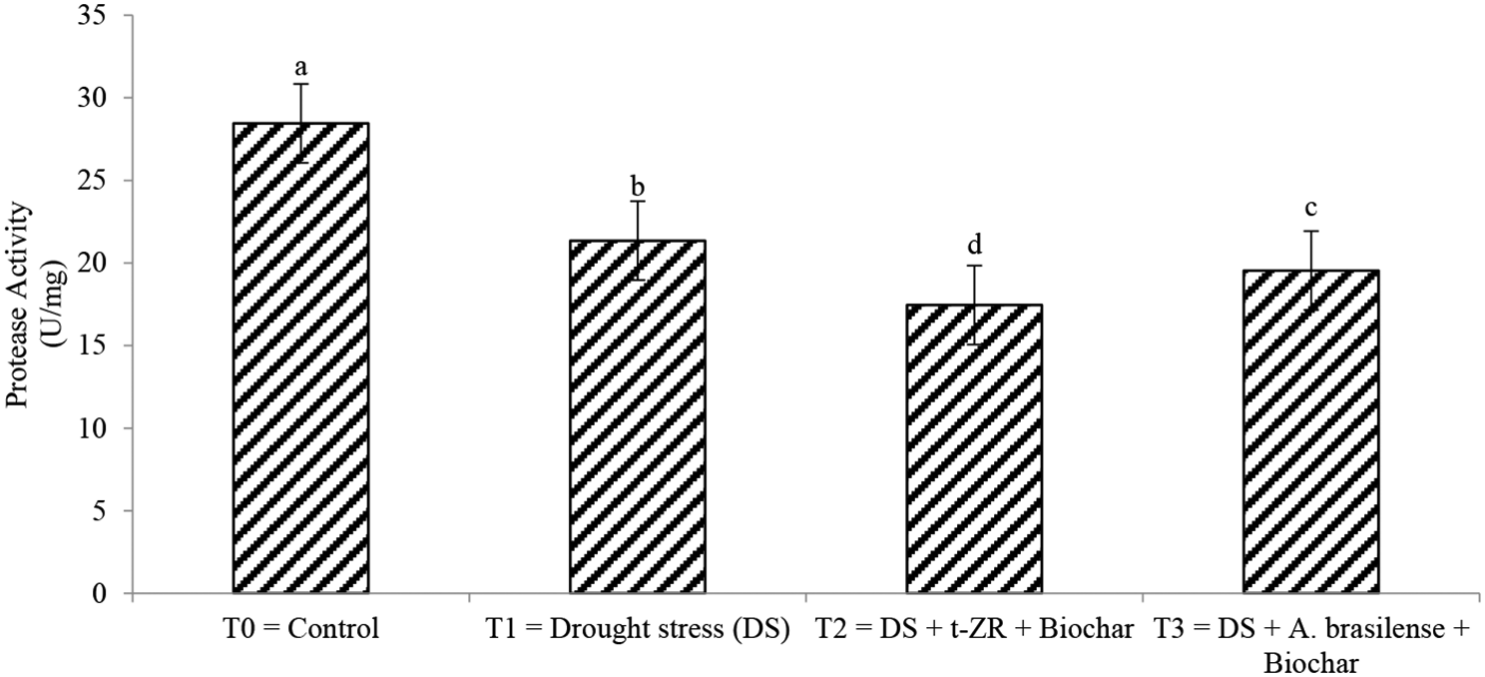
Effect of combine application of biochar with trans-zeatin riboside and with *Azospirillum brasilense* on protease activity (U/mg) of wheat under drought stress

Amylase and protease activity significantly decreased with the application of biochar with t-ZR and biochar with *A. brasilense*. The highest amylase activities were noticed in T_0_ (9.34 µmol/min/mg) followed by T_1_ (5.56 µmol/min/mg) and the lowest amylase activities (3.45 µmol/min/mg) were seen in T_2_ (Fig. 5). Highest protease activities were noticed in T_0_ (28.45 U/mg) under control treatments followed by T_1_ (21.35 U/mg) when drought stress conditions occurred having no application and lowest protease activities were noticed in T_2_ (17.45 U/mg) when biochar was applied with t-ZR (Fig. 6).

**Fig. 7:**
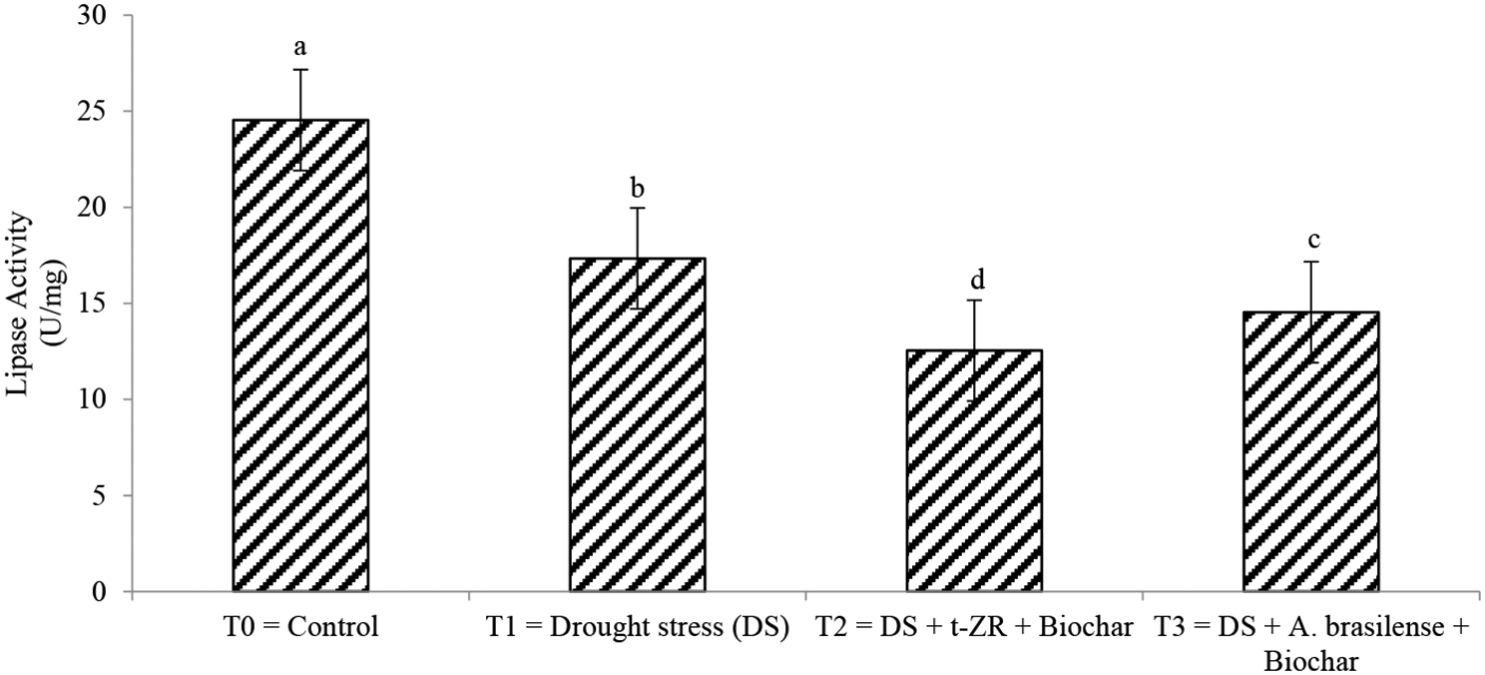
Effect of combine application of biochar with trans-zeatin riboside and with *Azospirillum brasilense* on lipase activity (U/mg) of wheat under drought stress

**Fig. 8:**
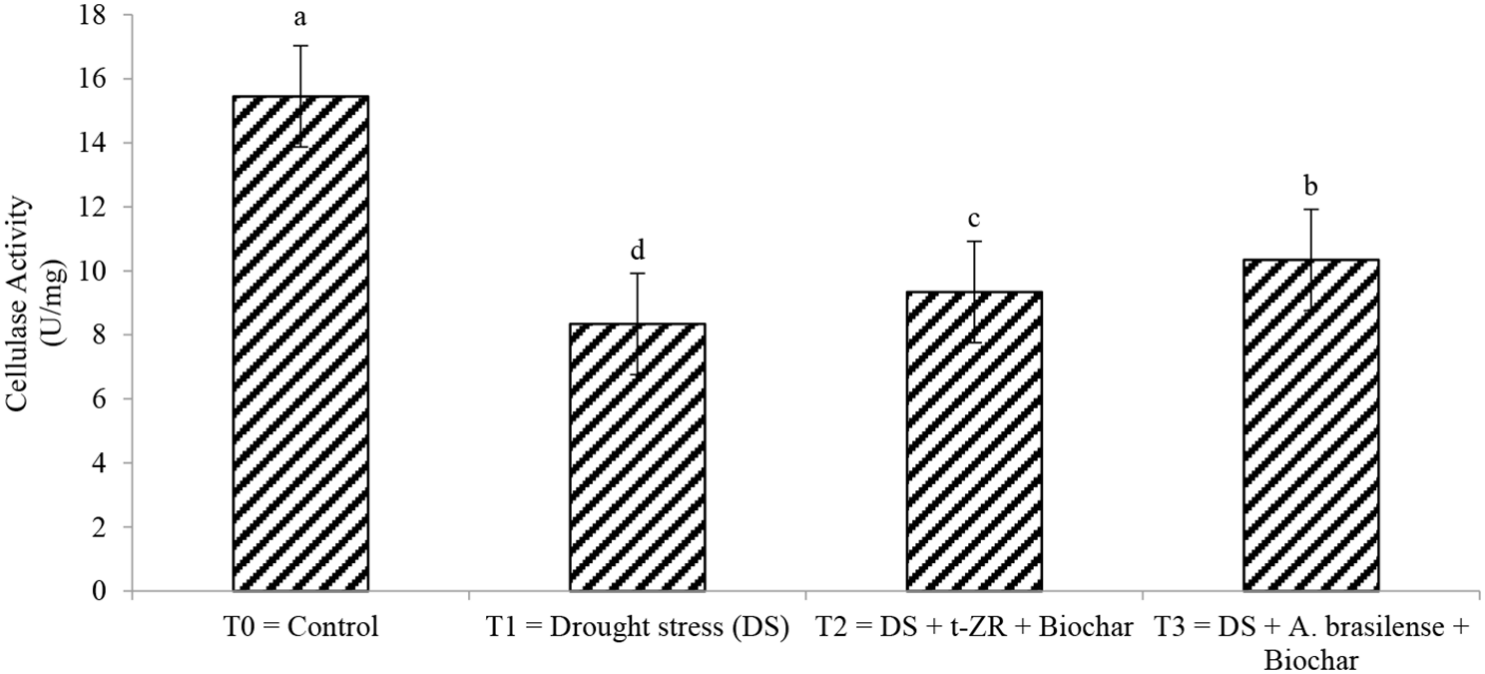
Effect of combine application of biochar with trans-zeatin riboside and with *Azospirillum brasilense* on cellulase activity (U/mg) of wheat under drought stress

Lipase activities significantly decreased with the application of biochar with t-ZR and biochar with *A. brasilense* of wheat under drought stress (Fig. 7). Highest lipase activities (24.54 U/mg) were noticed in T_0_ under control treatment followed by T_1_ (17.34 U/mg) when drought stress was applied and lowest lipase activities (12.54 U/mg) were seen in T_2_ when biochar was applied with t-ZR. The highest cellulase activities (15.45 U/mg) were noticed in T_0_ followed by T_3_ (10.34 U/mg) and the lowest cellulase activities (09.34 U/mg) were seen in T_1_ (Fig. 8).

**Fig. 9:**
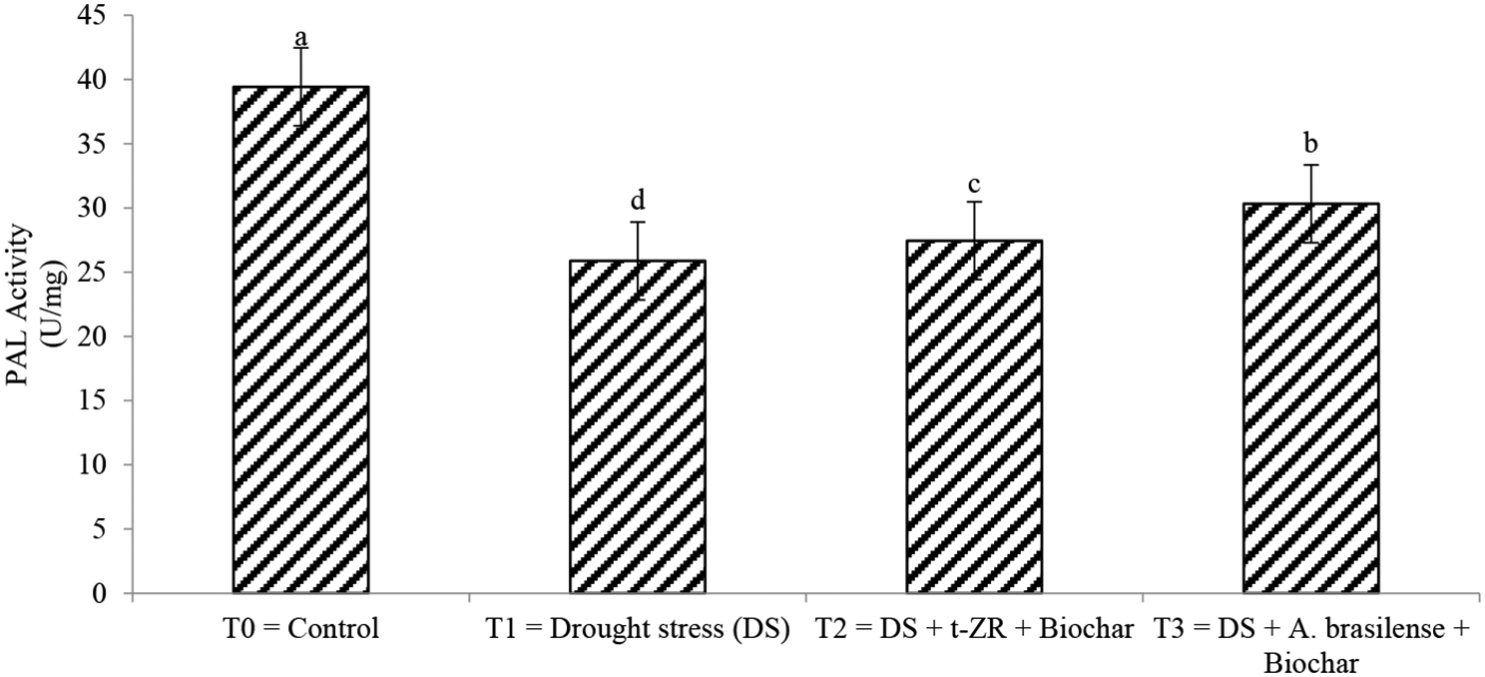
Effect of combine application of biochar with trans-zeatin riboside and with *Azospirillum brasilense* on phenylalanine ammonia lyase (PAL) (U/mg) of wheat under drought stress

**Fig. 10:**
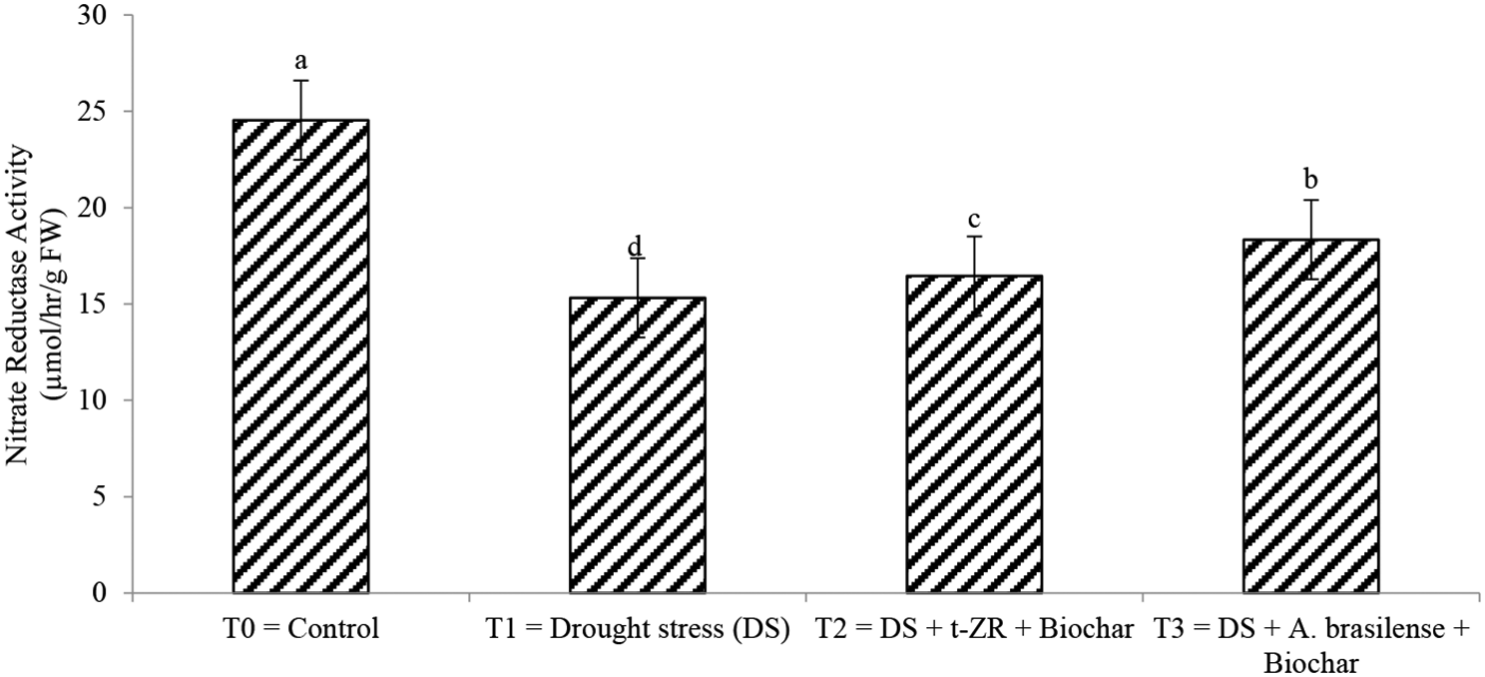
Effect of combine application of biochar with trans-zeatin riboside and with *Azospirillum brasilense* on nitrate reductase activity (µmol/hr/g FW) of wheat under drought stress

Phenylalanine ammonia-lyase (PAL) and nitrate reductase activities were significantly enhanced with the application of biochar with t-ZR and biochar with *A. brasilense* under drought stress (Figs. 9 and 10). The highest PAL (39.34 U/mg) and nitrate reductase activities (24.54 µmol/hr/g FW) were noticed in T_0_ under control treatment having no drought and no application followed by T_3_ (PAL: 30.32 U/mg, nitrate reductase: 18.34 µmol/hr/g FW) and lowest results were seen in T_1_ (PAL: 25.87 U/mg, nitrate reductase: 15.32 µmol/hr/g FW).

**Fig. 11:**
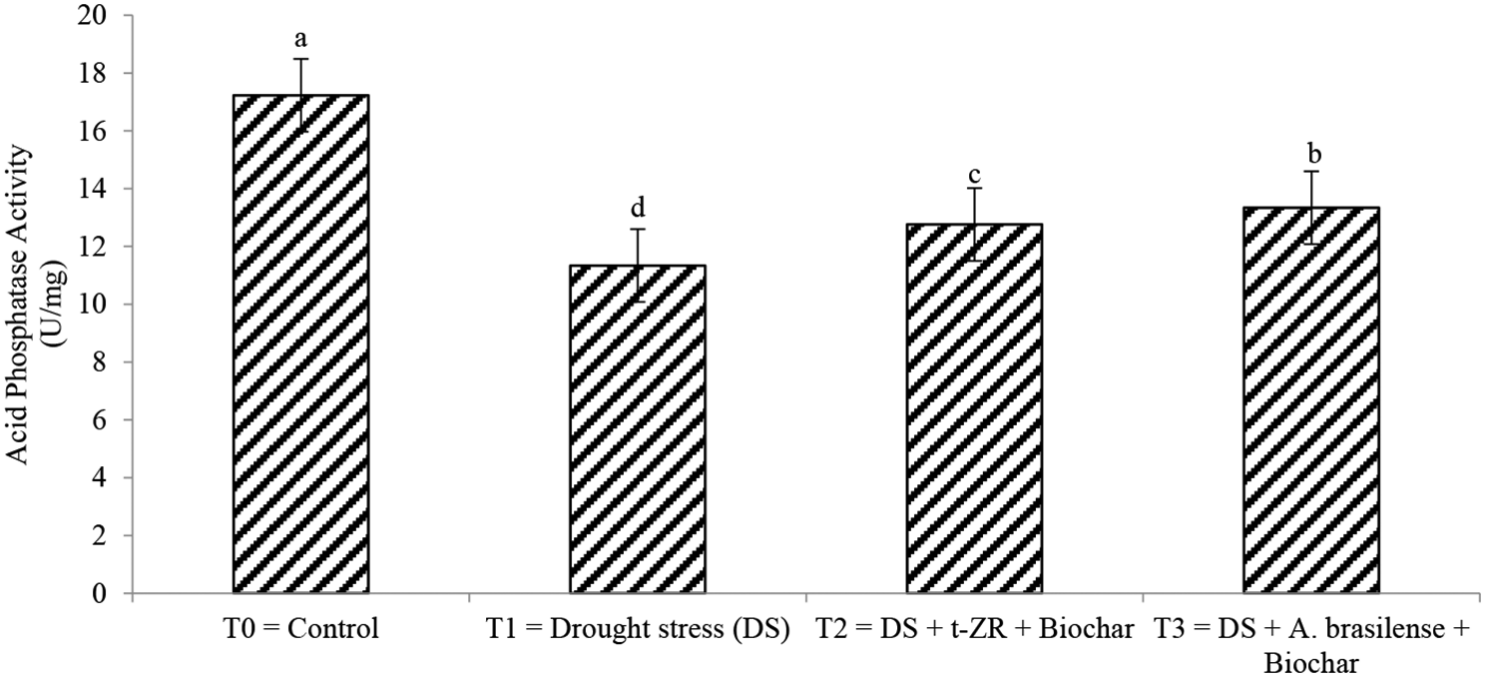
Effect of combine application of biochar with trans-zeatin riboside and with *Azospirillum brasilense* on acid phosphatase activity (U/mg) of wheat under drought stress

**Fig. 12:**
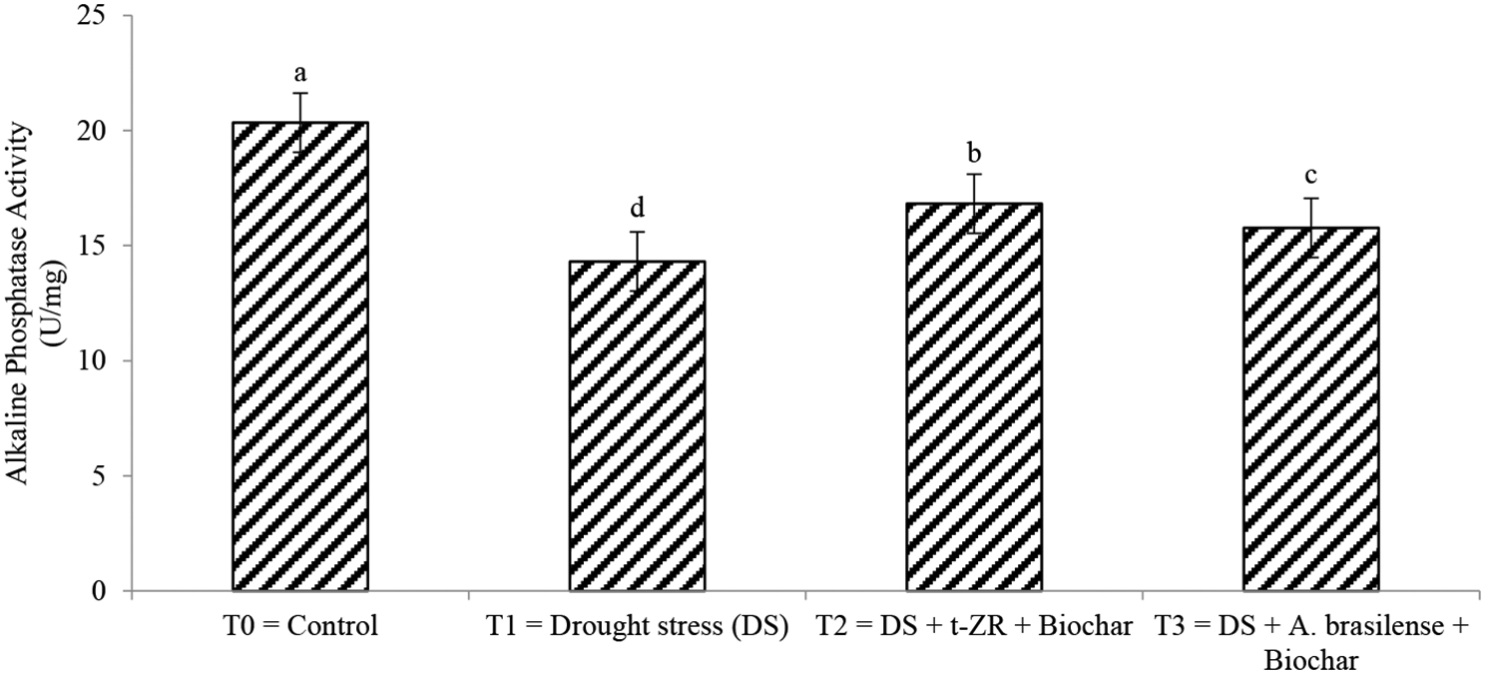
Effect of combine application of biochar with trans-zeatin riboside and with *Azospirillum brasilense* on alkaline phosphatase activity (U/mg) of wheat under drought stress

Acid phosphatase and alkaline phosphatase activities significantly improved with the application of biochar with t-ZR and biochar with *A. brasilense* under drought-stress conditions (Figs. 11 and 12). Highest acid phosphatase activities were noticed in T_0_ (17.23 U/mg) under control treatment followed by T_3_ (13.34 U/mg) when biochar was applied with *A. brasilense* under drought stress conditions and the lowest acid phosphatase activities were observed in T_1_ (11.34 U/mg) when drought stress was applied having no biochar application highest alkaline phosphatase activities were noticed in T_0_ (20.34 U/mg) followed by T_2_ (16.82 U/mg) and lowest alkaline phosphatase activities were seen in T_1_ (14.31 U/mg).

Soil dehydrogenase (DH) and soil urease (UR) were significantly affected by the application of biochar with t-ZR and biochar with *A. brasilense* under drought stress (Table 3). Highest DH (68.32 nmol TPF g^-1^ soil h^-1^) and UR (23.45 µmol NH_3_-N g^-1^ h^-1^) activities were noticed in T_0_ (DH: 63.45 nmol TPF g^-1^ soil h^-1^, UR: 21.45 µmol NH_3_-N g^-1^ h^-1^) followed by T_3_ and lowest DH (57.34 nmol TPF g^-1^ soil h^-1^) and UR (17.34 µmol NH_3_-N g^-1^ h^-1^) was observed in T_1_. Soil Acid Phosphatase (AcP) and Soil β-Glucosidase (βG) significantly increased with the application of biochar with t-ZR and biochar with *A. brasilense* under drought stress conditions. Highest AcP (30.46 µmol p-Nitrophenol g^-1^ h^-1^) and βG (6.28 µmol p-Nitrophenol g^-1^ h^-1^) activities were noticed in T_0_ followed by T_3_ (AcP: 27.94 µmol p-Nitrophenol g^-1^ h^-1^, βG: 4.67 µmol p-Nitrophenol g^-1^ h^-1^) and lowest AcP (22.34 µmol p-Nitrophenol g^-1^ h^-1^) and βG (2.45 µmol p-Nitrophenol g^-1^ h^-1^) activities were observed in T_2_.

**Table 3:** Effect of combine application of biochar with trans-zeatin riboside and with *Azospirillum brasilense* on soil dehydrogenase (DH), soil urease (UR), soil acid phosphatase (AcP), soil β-Glucosidase (βG) of wheat under drought stress

	Soil Dehydrogenase (DH) (nmol TPF g^− 1^ soil h^− 1^)	Soil Urease (UR) (µmol NH_3_-N g^− 1^ h^− 1^)	Soil Acid Phosphatase (AcP) (µmol p-Nitrophenol g^− 1^ h^− 1^)	Soil β-Glucosidase (βG) (µmol p-Nitrophenol g^− 1^ h^− 1^)
T_0_ = Control	68.32 a (± 0.3301)	23.45 a (± 0.4346)	30.46 a (± 0.3514)	6.28 a (± 0.4024)
T_1_ = Drought stress (DS)	57.34 d (± 0.3820)	17.34 d (± 0.6165)	22.34 d (± 0.3296)	2.45 d (± 0.2419)
T_2_ = DS + t-ZR + Biochar	60.45 c (± 0.2927)	18.45 c (± 0.5107)	25.36 c (± 0.3084)	3.72 c (± 0.4419)
T_3_ = DS + *A. brasilense +* Biochar	63.45 b (± 0.3604)	21.45 b (± 0.4988)	27.94 b (± 0.3871)	4.67 b (± 0.4083)

Different letters with the data shows statistical difference between the treatments

SOC (soil organic C), DOC (dissolved organic C), and DON (dissolved organic N) were significantly enhanced with the application of biochar with t-ZR and biochar with *A. brasilense* under drought stress conditions. Highest SOC (1.64 g kg^-1^), DOC (149 µg g^-1^) and DON (23.45 µg g^-1^) were noticed in T_0_ followed by T_3_ (SOC: 1.45 g kg^-1^, DOC: 139 µg g^-1^, DON: 20.32 µg g^-1^) and lowest SOC (1.12 g kg^-1^), DOC (128 µg g^-1^) and DON (16.56 µg g^-1^) were noticed in T_1_ (Table 4).

**Table 4:** Effect of combine application of biochar with trans-zeatin riboside and with *Azospirillum brasilense* on SOC (soil organic C), DOC (dissolved organic C), DON (dissolved organic N) of wheat under drought stress

	SOC (g kg^− 1^)	DOC (µg g^− 1^)	DON (µg g^− 1^)
T_0_ = Control	1.64 a (± 0.2204)	149 a (± 0.3650)	23.45 a (± 0.3481)
T_1_ = Drought stress (DS)	1.12 d (± 0.4038)	128 d (± 0.3937)	16.56 d (± 0.3354)
T_2_ = DS + t-ZR + Biochar	1.32 c (± 0.3175)	134 c (± 0. 3210)	18.37 c (± 0.3173)
T_3_ = DS + *A. brasilense +* Biochar	1.45 b (± 0.3912)	139 b (± 0. 2631)	20.32 b (± 0.4950)

Different letters with the data shows statistical difference between the treatments

## Discussion

Drought stress is a main abiotic stress that can reduction in wheat crop yield especially in under developing countries, Use of biochar, trans-zeatin riboside (t-ZR) and *Azospirillum brasilense* in crop production is very helpful to get sustainable wheat crop yield under such kind of the conditions. Applying biochar containing trans-zeatin riboside (t-ZR) and *Azospirillum brasilense* to wheat crops had a significant effect on peroxidase activities in wheat plants [16]. Biochar enhances the structure of the soil by boosting microbial activity, nutrient availability, and water retention in the soil [6]. A plant hormone known as t-ZR is a cytokinin that influences numerous physiological processes linked to growth and stress response. It may also have an extra impact on peroxidase activity [15]. *A. brasilense* increases plant growth by enhancing nutrient uptake and possibly influencing the regulation of peroxidase activity [34]. *A. brasilense*, t-ZR, and biochar application boost peroxidase activity under drought stress [15]. The combined actions of A. brasilense and biochar produced a favorable microbiological environment that supported plant physiological processes during drought stress, which is why the applied treatment boosted peroxidase activity. Because each treatment has a different method of action and affects enzyme activities under drought stress, the control plants showed higher peroxidase activity than the biochar + t-ZR and biochar + Azospirillum-treated plants. Plant growth under stress is impacted by t-ZR, whereas soil water retention and microbial activity are enhanced by biochar. Working together, *A. brasilense* may also help plants become more drought-tolerant; this tactic may increase wheat’s peroxidase levels and fortify the plant’s defenses against stress brought on by drought [35]. Zaheer et al. [6] reported that biochar increases soil water retention and microbiological activity. Plants that are under stress may benefit from the cytokinin t-ZR [15]. Ali et al. [35] reported that *A. brasilense* enhances nutrient availability and fosters plant development. The two vital enzymes that scavenge reactive oxygen species (ROS), superoxide dismutase and catalase are impacted by the combination of these elements [36]. The cooperative effect may increase antioxidant enzyme activity, enhancing the wheat plant’s resistance to oxidative stress caused by dryness [37]. Regarding a number of relevant studies, the findings derived from the research on Carex duriuscula by Hou et al. [38], wheat plants under stress by Caverzan et al. [39], bread wheat by Qayyum et al. [40], and drought-exposed wheat cultivars by Huseynova [41] provide important new understandings of the mechanisms underlying plant enzymatic responses and drought tolerance. All of these studies point to the importance of gaseous exchange, osmotic adjustment, osmolyte synthesis, and antioxidant enzyme activity in improving plant resistance to water stress. Our study supports these results by showing how biochar, t-ZR, and A. brasilense work together to enhance enzyme activities in water-stressed wheat plants, including peroxidase, catalase, superoxide dismutase, polyphenol oxidase, and amylase. This emphasizes the significance of novel methods like biochar and microbial inoculants in mitigating the negative impacts of water stress.

Significant effects on important physiological processes and enzymatic activity are observed in wheat when biochar, t-ZR, and *A. brasilense* are applied, particularly when drought stress is present [15]. Biochar enhances soil structure and microbial activities which is very helpful for a suitable environment for plant growth. Polyphenol oxidase (PPO) expression is frequently altered by microbial interactions, so biochar affects PPO activities. Polyphenol oxidase (PPO) is an essential enzyme in the plant’s defense mechanism against abiotic stress and its production is improved with the application of biochar and *A. brasilense* [42]. t-ZR is associated with several physiological processes, including cell division and differentiation, which impact the activity of the enzyme amylase and are involved in starch metabolism [15]. Enzymatic activities that are essential for metabolic processes are impacted when wheat grows using biochar containing t-ZR and biochar containing *A. brasilense* during drought [43]. By altering the microbial communities in the soil and the availability of nutrients, biochar may affect the activities of lipase, cellulase, and proteases [44]. It’s possible that t-ZR can regulate these enzymatic activities because of its function in plant growth and stress responses [15]. The growth-promoting bacteria *A. brasilense* for plants increases the availability of nutrients, which in turn influences the activities of lipase, protease, and cellulose enzymes crucial for nutrient uptake and cell wall modification [45].

Phenylalanine ammonia-lyase (PAL) and nitrate reductase activities significantly increased in response to both biochar compositions, showing a favorable response to the mitigation of drought stress [6]. Addition of the biochar in the soil can enhance the soil’s ability to retain moisture and release nutrients that directly improve the plant metabolic processes [42]. The observed increase in PAL and nitrate reductase activities are most likely the result of the combined effects of t-ZR and A. brasilense, which are known to stimulate plant growth and metabolic pathways [15]. When biochar was applied having no drought stress in wheat PAL and nitrate reductase activities can increase. This bolsters the hypothesis that plants react physiologically to drought and other stressors and that using biochar can mitigate those effects [46]. The lowest levels of PAL and nitrate reductase activities were observed where drought stress was applied without the use of biochar supplementation. This result highlights the critical role that biochar plays in enhancing plant stress tolerance. Activities of alkaline phosphatase and acid phosphatase can increased with the application of biochar as it can increase the soil fertility, organic matter, and physiological processes of the plant that directly increase the photosynthates and growth hormones in the plant [47]. Duff et al. [48] reported that the acid phosphatase is associated with the mobilization of phosphorus, whereas alkaline phosphatase facilitates the uptake of nutrients. The increase in these enzyme activities suggests that the biochar amendments may have contributed to the improved nutrient availability in the root zone [49].

Significant increases in soil dehydrogenase and urease activities have been noticed by biochar and *A. brasilense* that shows the biochar efficacy in enhancing these enzyme functions in drought-stressed wheat-growing soil [4, 6]. Biochar that contained t-ZR exhibited decreased activity for both enzymes, indicating that in similar conditions, it might not have as much of an impact on urease and dehydrogenase [50]. The application of both biochar formulations significantly increased soil β-glucosidase (βG) and soil acid phosphatase (AcP) activities under drought stress conditions [6]. The control treatment showed the highest activities for AcP and βG indicating that the absence of biochar supplementation resulted in the most notable increase in these enzyme activities. Zaheer et al. [6] reported that the biochar infused with *A. brasilense* exhibited noteworthy activity for both AcP and βG, indicating its potential utility in augmenting these soil enzymatic processes. Reduced activities for AcP and βG were observed in biochar containing t-ZR [49], suggesting that drought stress may not have as much of an impact on these enzyme activities. Biochar with *A. brasilense* and Biochar with t-ZR both have a positive effect on soil properties especially for the SOC, DOC, and DON. Dong et al. [51, 52] reported similar findings with the application of biochar. SOC promotion is necessary to maintain soil fertility and structure [4]. Both biochar infused with t-ZR and biochar infused with *A. brasilense* support this attribute. The dissolved organic carbon (DOC) levels analysis demonstrates the advantageous effects of both biochar formulations. Elevated DOC levels can be used as a marker for increased microbial activity, the decomposition of organic matter, and the availability of nutrients. Biochar containing t-ZR and biochar containing *A. brasilense* are both effective in fostering a favorable environment for DOC [37] although the specific mechanisms enhancing dissolved organic carbon (DOC) in the soil may differ. Dissolved organic nitrogen (DON) shows that both biochar formulations have a positive effect on the nitrogen dynamics in the soil. An indication of better plant nutrient availability is elevated DON levels. Their respective efficacies show that biochar containing t-ZR and biochar containing *A. brasilense* can both increase the amount of dissolved organic nitrogen in soil used for growing wheat during drought stress [6, 53].

## Conclusion

Drought stress significantly damaged the enzymatic activities of wheat plants and soil properties. Biochar with trans-Zeatin Riboside and with *Azospirillum brasilense* both are very effective for plant growth promotion and to increase enzymatic activities under drought stress conditions. The most promising combination for mitigating the detrimental effects of drought stress on enzymatic activity was *A. brasilense* with biochar. *A. brasilense* and biochar can enhance the enzymatic and soil structure which may be helpful for water-scarce environments that are very effective for sustainable agriculture. So it is recommended to for the farmers to use the *A. brasilense* with biochar to improve wheat growth especially under drought stress condition.

### Electronic supplementary material

Below is the link to the electronic supplementary material.

Supplementary Material 1

## Data Availability

Data is provided within the manuscript or supplementary information files.
